# Deepath-SCC: a deep learning model for accurate tissue origin identification in squamous cell carcinoma

**DOI:** 10.1038/s41698-026-01405-1

**Published:** 2026-04-10

**Authors:** Siwei Lu, Yue Pang, Huer Wen, Linyi Hu, Xiaoli Zhou, Xiao Yang, Xiaowei Qi, Cong Liu, Jing Liu, Peng Qi, Shenglin Huang, Qinghua Xu, Yifeng Sun, Qifeng Wang

**Affiliations:** 1 Department of Pathology, Fudan University Shanghai Cancer Center, and Shanghai Key Laboratory of Medical Epigenetics, Institutes of Biomedical Sciences, Fudan University, Shanghai, China; 2 Department of Oncology, Shanghai Medical College, Fudan University, Shanghai, China; 3 Institute of Pathology, Fudan University, Shanghai, China; 4 Canhelp Genomics Research Center, Canhelp Genomics Co. Ltd., Hangzhou, China; 5 College of Computer Science and Technology, Tongji University, Shanghai, China; 6 Urology & Nephrology Center, Department of Urology, Zhejiang Provincial People’s Hospital, Affiliated People’s Hospital, Hangzhou Medical College, Hangzhou, Zhejiang China; 7 Department of Pathology, The Third Affiliated Hospital of Nanjing Medical University, Changzhou, China; 8 Department of Pathology, Affiliated Hospital of Jiangnan University, Wuxi, China; 9 Center of Medical Physics, Nanjing Medical University, Changzhou, China

**Keywords:** Cancer, Computational biology and bioinformatics, Oncology

## Abstract

Squamous cell carcinoma (SCC) is a common malignancy that arises in diverse organs and often exhibits overlapping histological and immunophenotypic features, making accurate determination of the tissue of origin challenging with conventional pathology. To address this, we developed Deepath-SCC, a deep learning model for pan-SCC tissue origin identification directly from hematoxylin and eosin-stained whole-slide images. A retrospective cohort of 4217 whole slide images across nasopharyngeal, head and neck/esophageal, lung, cervical, and urothelial carcinomas was assembled for model training and validation. In the internal test set, Deepath-SCC reached accuracy of 91.2%, with micro area under the receiver operating characteristic curves (AUROC) reaching 0.986 (95% CI: 0.984–0.989). Among high-confidence predictions (similarity score ≥0.7914), the overall accuracy increased to 96.2%, including 96.4% for primary SCCs and 94.4% for metastatic SCCs. In an external test set, Deepath-SCC achieved an accuracy of 86.1% with an AUROC of 0.972 (95% CI: 0.961–0.982). These results provide initial evidence supporting the feasibility of deep learning–based digital pathology for tissue-of-origin prediction in pan-SCC. Deepath-SCC represents an efficient, and cost-effective computational approach that may complement existing diagnostic workflows, particularly in challenging or resource-limited clinical settings.

## Introduction

Squamous cell carcinoma (SCC) is one of the most common malignancies, arising from the epithelial of the aerodigestive and genitourinary tracts^1,2^. While non-SCC tumors from different organs exhibit certain site-specific histological and immunophenotypic features, SCCs frequently share a convergent histopathological feature, characterized by positive expression of squamous differentiation markers^3^. These markers are diagnostically useful for distinguishing SCC from non-SCC, but are insufficient for determining the tissue of origin.

The morphological and immunophenotypic similarities of SCCs across sites pose significant diagnostic challenges, particularly in cancers of unknown primary (CUP), where SCC accounts for approximately 17–22% of cases in China^4,5^. Evidence from the Fudan CUP-001 trial showed that site-specific therapy guided by a 90-gene expression assay significantly improved progression-free survival (PFS) compared with empirical chemotherapy, especially among patients with SCC (hazard ratio = 0.26)^5^.

Viral biomarkers such as human papillomavirus (HPV) and Epstein-Barr virus (EBV) are employed to aid in identifying the primary sites of cervical and nasopharyngeal SCC, respectively^1,6^. However, the presence of HPV-related SCCs in the oropharynx and EBV-associated lymphoepithelioma-like carcinomas in multiple organs limits their specificity as origin indicators^7,8^. Molecular profiling approaches, including transcriptomic, genomic, and epigenomic analyses have been successfully employed in tissue-of-origin prediction in pan-cancer cohorts^9–11^. However, the clinical applicability of many existing molecular approaches remains limited by high costs, complex workflows, and reliance on specialized instruments, particularly in low-resource settings. In parallel, recent advances in artificial intelligence, particularly large-scale foundation models, have shown promise in histopathological image analysis^12–14^. In the domain of cutaneous SCC, deep learning–based systems have been developed for tasks such as tumor detection, histological grading, and prediction of progression risk^15–18^. Despite these advances, their utility in classifying pan-SCC tissues of origin, especially in metastatic settings, has not been systematically evaluated.

To address this unmet need, we developed and validated a deep learning-based model for pan-SCC tissue-of-origin classification. Our aim is to provide an accurate, cost-effective, and interpretable diagnostic tool to improve clinical decision-making and personalized treatment for patients with pan-SCCs.

## Results

### Dataset description

In this study, we collected a total of 4217 hematoxylin and eosin (H&E) -stained Whole slide images (WSIs) of SCCs along with corresponding clinicopathological information from Fudan University Shanghai Cancer Center (FUSCC), Changzhou No.2 People’s Hospital (CPH), also affiliated to Nanjing Medical University, and The Cancer Genome Atlas (TCGA). These samples encompass five major types of high-incidence SCCs: cervical squamous cell carcinoma (CSCC) (n = 1433), head and neck or esophageal squamous cell carcinoma (HNE) (n = 956), lung squamous cell carcinoma (LUSCC) (n = 939), nasopharyngeal carcinoma (NPC) (n = 198), and urothelial carcinoma (UC) (n = 691) (Table 1). Among them, 3578 cases (84.8%) are primary tumors and 639 cases (15.2%) are metastatic tumors. Overall, 2267 WSIs are assigned to the training set for self-supervised model development, and 1950 to the internal test set. The independent external test set obtained from the Affiliated Hospital of Jiangnan University (AHJU) comprises 209 WSIs, including 52 CSCC, 66 HNE, 39 LUSCC, 32 NPC, and 20 UC WSIs.

**Table 1:** Clinicopathological features in the dataset

Parameter	Overall n=4217	Training set n=2267	Internal test set n=1950	Internal test set n=1662 (Tiles≥20)
Gender				
Male	2243	1150	1093	925
Female	1974	1117	857	737
Age				
<60	1933	1089	844	735
≥60	2275	1175	1100	921
Unknown	9	3	6	6
Primary	3578	1880	1698	1460
Metastasis	639	387	252	202
Tumor site				
Cervix	1433	854	579	528
Head and neck/esophagus	956	490	466	402
Lung	939	486	453	397
Nasopharynx	198	121	77	59
Urinary tract	691	316	375	276
Tumor differentiation				
Well	45	28	17	17
Well-moderate	69	43	26	22
Moderate	532	313	219	202
Moderate-poorly	327	189	138	126
Poorly	782	473	309	261
Undifferentiated	106	68	38	27
Unknown	2356	1153	1203	1007

### Performance of the Deepath-SCC model

**Fig. 1:**
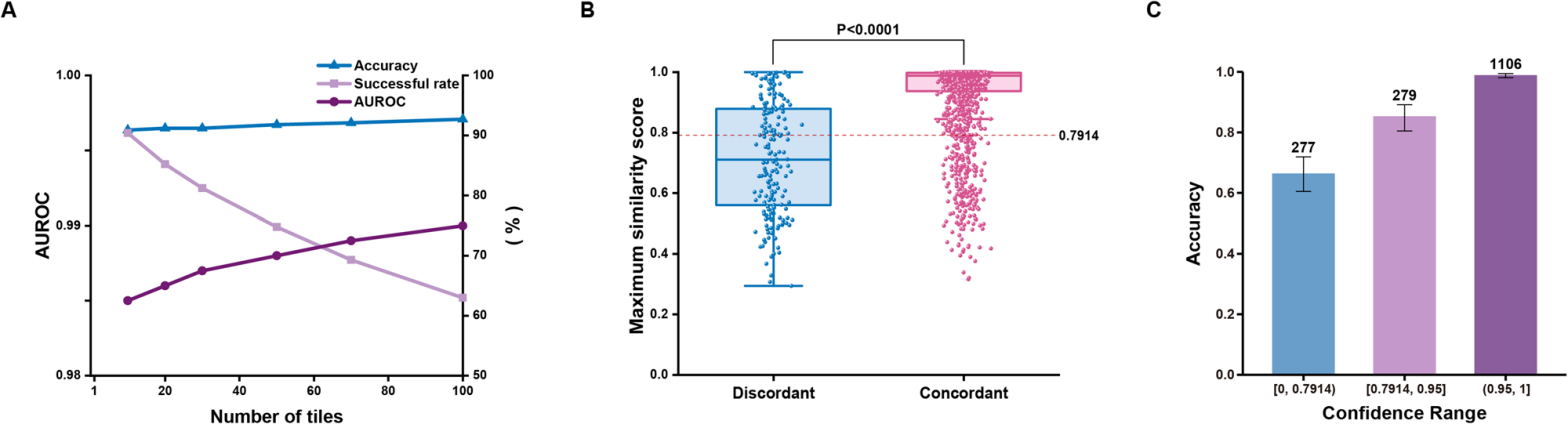
Subtype composition and performance evaluation of the model in internal test set. **A** The accuracy, the micro-AUROC, and the successful rate under different numbers of tiles. **B** Distribution of maximum similarity scores in discordant and concordant groups. **C** The classification accuracy across three confidence intervals defined by maximum similarity scores.

To enhance model stability and classification reliability, model performance was systematically evaluated under different tumor tiles count thresholds (Table S1, Fig. S1). A minimum threshold of 20 tumor tiles per sample was subsequently established. Analysis of the internal test set demonstrated that when this criterion was met, the model achieved robust performance, with a micro-area under the receiver operating characteristic curve (AUROC) of 0.986 (95% CI: 0.984–0.989) and a patient-level classification success rate of 85.2% (1662/1950; Fig. 1A). This subset included 528 CSCC, 402 HNE, 397 LUSCC, 59 NPC, and 276 UC WSIs (Table 1). Among these 1662 WSIs, 1460 (87.8%) were primary tumors, including 424 CSCC, 367 HNE, 371 LUSCC, 38 NPC, and 260 UC WSIs, whereas 202 (12.2%) were metastatic tumors, including 104 CSCC, 35 HNE, 26 LUSCC, 21 NPC, and 16 UC WSIs. Under this condition, the overall classification accuracy reached 91.2% (1516/1662), with accuracies of 92.1% (1344/1460) for primary tumors and 85.1% (172/202) for metastatic tumors (Fig. S2A).

To optimize the clinical utility of the Deepath-SCC model, we performed an optimal binning analysis using SPSS on samples with ≥20 tumor tiles. This analysis identified a threshold of 0.7914 for the maximum similarity score. As shown in Fig. 1B, cases with concordant predictions and reference diagnoses exhibited significantly higher similarity scores than discordant cases (median 0.988 vs 0.711; p < 0.0001). Based on the derived threshold, cases were stratified into three confidence intervals: [0, 0.7914), [0.7914, 0.95], and (0.95, 1.0]. Classification accuracy increased substantially across these strata, from 66.4% (184/277) in the low-confidence group to 85.3% (238/279) and 98.9% (1094/1106) in the moderate- and high-confidence groups, respectively (Fig. 1C).

**Fig. 2:**
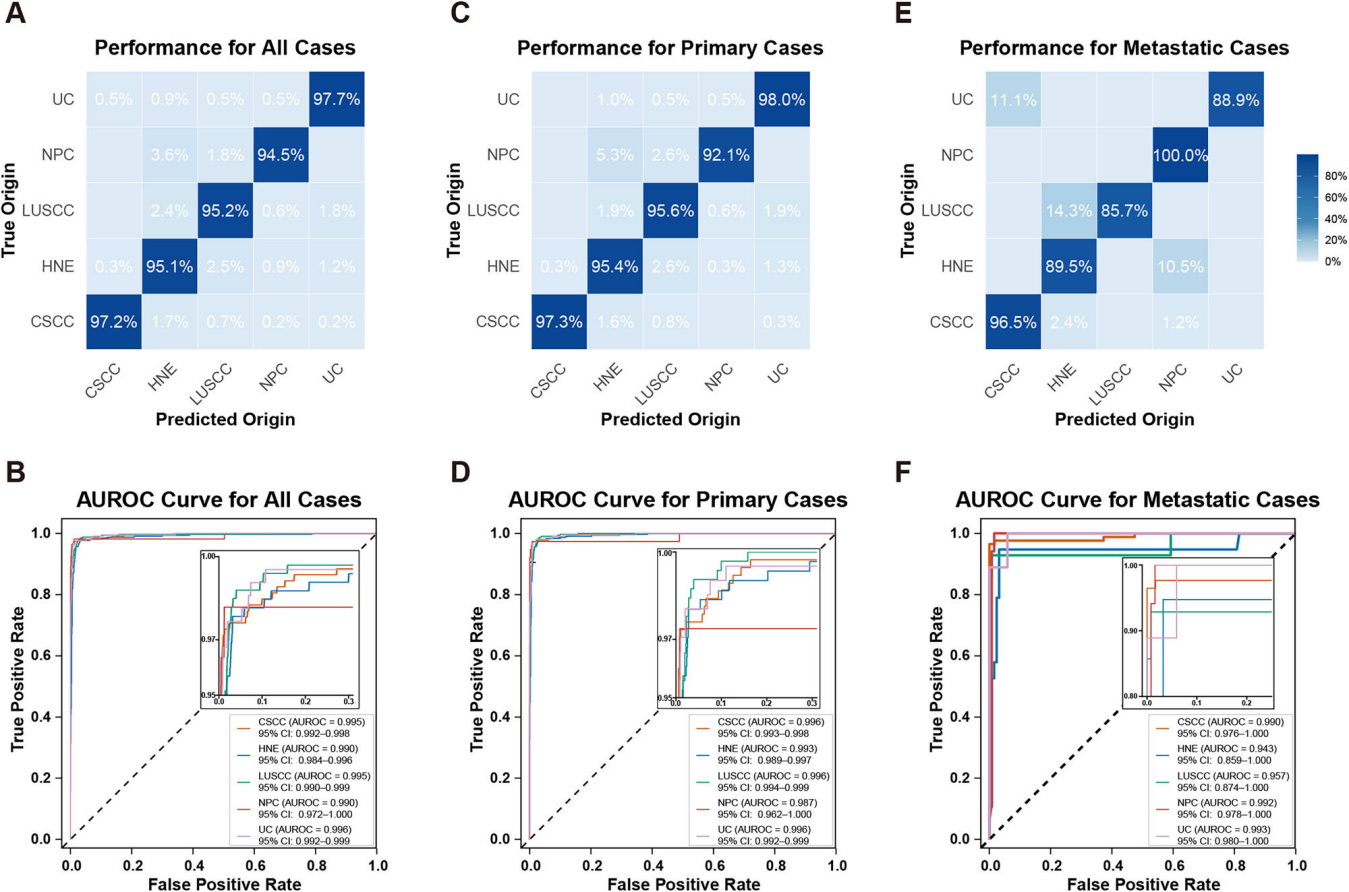
Deepath-SCC classification performance across all, primary, and metastatic squamous cell carcinoma in the internal test set. Confusion matrices showing overall prediction accuracy for (**A**) all cases, **C** primary cases, and **E** metastatic cases. The AUROC curves for (**B**) all cases, **D** primary cases, and **F** metastatic cases. The percentages for each group may not add to 100 due to rounding. CSCC cervical squamous cell carcinoma, HNE head and neck or esophageal squamous cell carcinoma, LUSCC lung squamous cell carcinoma, NPC nasopharyngeal carcinoma, UC urothelial carcinoma.

Applying dual filtering criteria of ≥ 20 tumor tiles and a similarity score threshold ≥ 0.7914, a total of 1385 WSIs were retained, including 1241 primary tumors (375 CSCC, 306 HNE, 317 LUSCC, 38 NPC, and 205 UC) and 144 metastatic tumors (85 CSCC, 19 HNE, 14 LUSCC, 17 NPC, and 9 UC). The model achieved an overall accuracy of 96.2% (1332/1385) (Fig. 2A), with AUROC values ranging from 0.990 for HNE and NPC to 0.996 for UC (Fig. 2B). The confusion matrix showing the numbers of correct and incorrect predictions is presented in Fig. S2B. Among primary SCCs, the model reached an accuracy of 96.4% (1196/1241), with subtype-specific accuracies ranging from 92.1% for NPC to 98.0% for UC, and AUROC values ranging from 0.987 (NPC) to 0.996 (CSCC, LUSCC, and UC) (Fig. 2C, D). In metastatic SCCs, the model achieved an accuracy of 94.4% (136/144), with subtype accuracies ranging from 85.7% (LUSCC) to 100.0% (NPC), and AUROC values from 0.943 (HNE) to 0.993 (UC) (Fig. 2E, F).

### Classification performance in the independent external test set

**Fig. 3:**
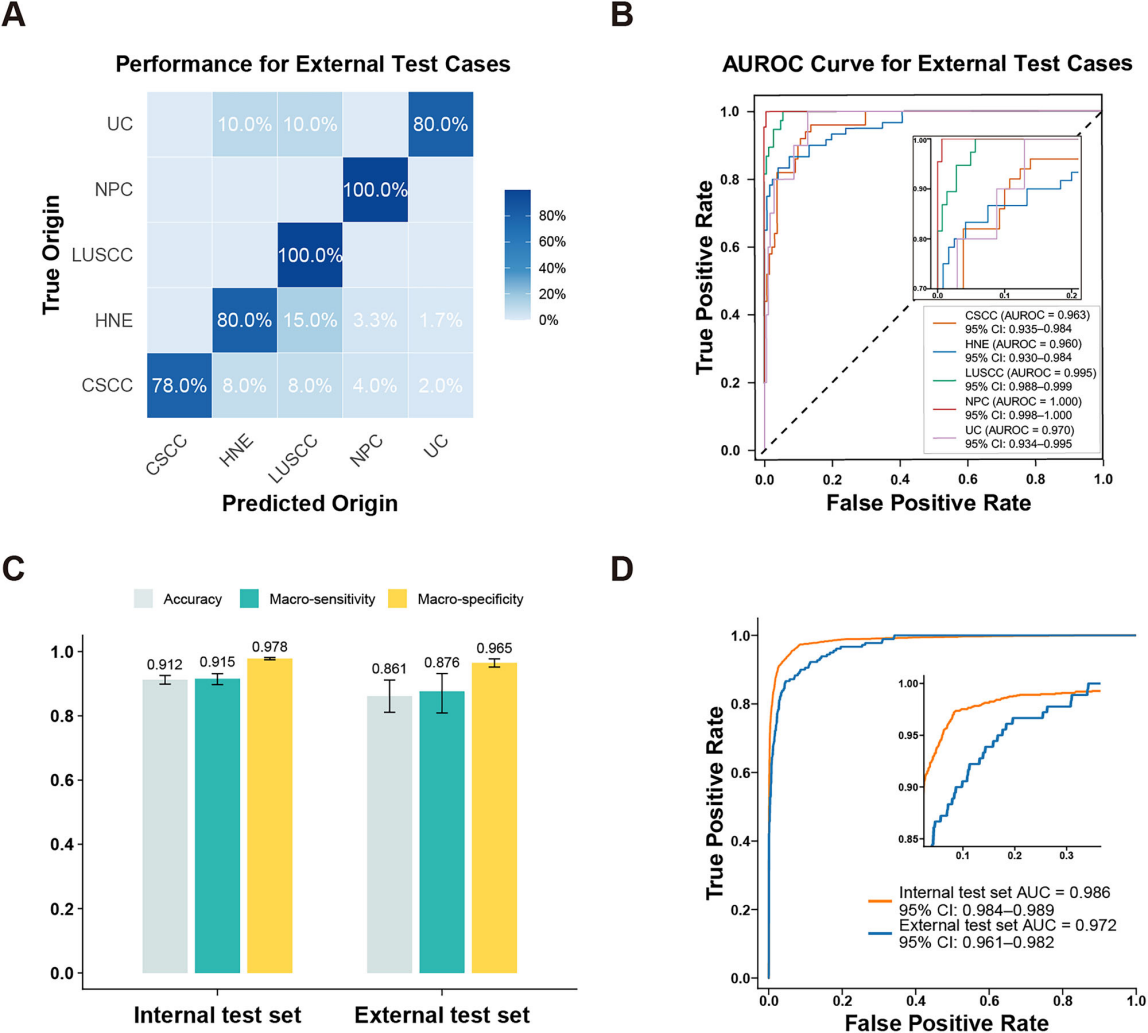
Performance of the Deepath-SCC model in the independent external test set. **A** Confusion matrix. **B** ROC curves. **C** Comparison of performance between the internal test set and the external test set. Error bars represent 95% confidence intervals. **D** Comparison of micro-AUROC between the internal test set and the external test set. CSCC cervical squamous cell carcinoma, HNE head and neck or esophageal squamous cell carcinoma, LUSCC lung squamous cell carcinoma, NPC nasopharyngeal carcinoma, UC urothelial carcinoma.

A total of 209 WSIs were initially included as the independent external test set. Among these, 180 WSIs met the inclusion criterion of at least 20 tumor tiles and were included for subsequent analyses. The retained cohort comprised 50 CSCC, 60 HNE, 38 LUSCC, 22 NPC, and 10 UC WSIs. In this independent external test set, Deepath-SCC model achieved an overall classification accuracy of 86.1% (155/180). Subtype-specific analyses indicated that classification accuracy reached 100.0% for both LUSCC and NPC (Fig. 3A). The overall micro-AUROC was 0.972 (95% CI: 0.961 - 0.982), with subtype-specific AUROC values ranging from 0.960 for HNE to 1.000 for NPC (Fig. 3B). Figure 3C compares the overall accuracy, macro-sensitivity, and macro-specificity between the internal test set and the external test set, while Fig. 3D shows the corresponding micro-AUROC values.

### Model interpretability and feature visualization

**Fig. 4:**
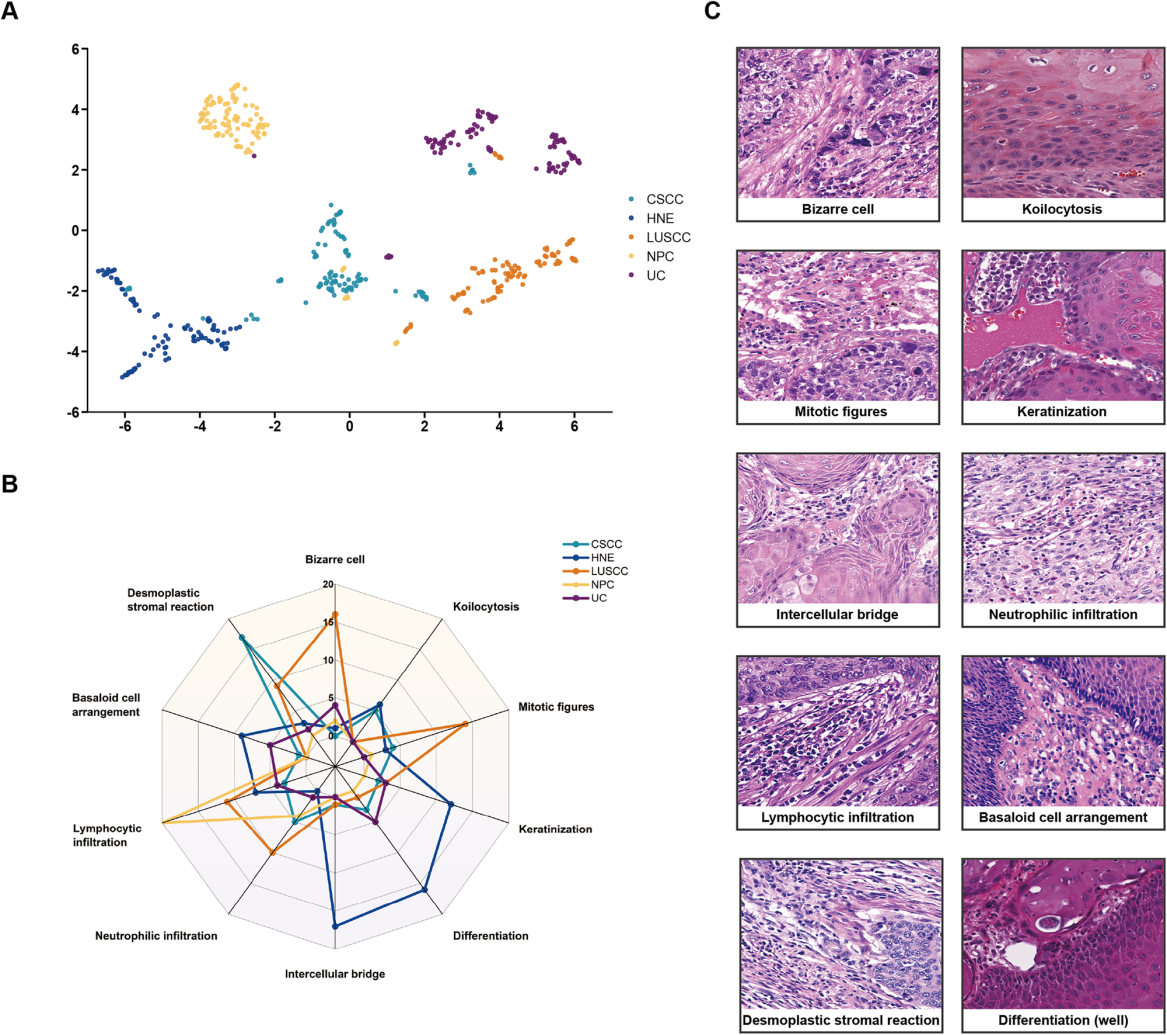
Morphological feature analysis based on representative high-confidence tiles. **A** UMAP visualization of high-dimensional features extracted from the top five tiles of top 20 concordant cases with the highest similarity scores from each SCC subtype. Each dot represents a single tile, colored by SCC subtype, illustrating distinct distribution patterns in the feature space. **B** Distribution of pathologist assessed scores for ten histological features that showed significant inter-subtype differences (p < 0.001). **C** Representative H&E-stained tile images corresponding to the ten key histological features. CSCC cervical squamous cell carcinoma, HNE head and neck or esophageal squamous cell carcinoma, LUSCC lung squamous cell carcinoma, NPC nasopharyngeal carcinoma, UC urothelial carcinoma.

To enhance interpretability and offer morphological insights into the model’s predictions, we selected the top 20 concordant cases with the highest similarity scores from each SCC subtype. For each case, the top five tiles with the highest tile scores were extracted and analyzed. These high-dimensional features were visualized using Uniform Manifold Approximation and Projection (UMAP), revealing distinct clustering patterns across the five SCC subtypes (Fig. 4A).

To further dissect subtype-specific morphological characteristics, two expert pathologists independently evaluated 11 histological features across the selected cases. Of these, ten features showed statistically significant differences among subtypes (Fisher’s exact test, p < 0.001; Table S2). Figure 4B summarizes the distribution of these ten discriminative features across SCC subtypes. CSCC was characterized by a pronounced desmoplastic stromal reaction (17/20). HNE showed the highest prevalence of keratinization (12/20) and intercellular bridges (17/20), clearly distinguishing this subtype from others in terms of squamous differentiation. LUSCC demonstrated a higher frequency of bizarre cells (16/20) and mitotic figures (14/20). NPC was characterized by marked lymphocytic infiltration (20/20), which was present in all evaluated cases. Representative histological images illustrating these features are shown in Fig. 4C.

### Comparison of Deepath-SCC and the 90-gene expression assay

To assess the performance of Deepath-SCC in the CUP, we selected 16 provisional CUP cases that met quality control criteria and had confirmed diagnoses, serving as an additional test set. All samples were analyzed using both the 90-gene expression assay and the Deepath-SCC model. Detailed clinicopathological features of 16 cases are provided in Table S3. The Deepath-SCC model produced predictions consistent with the final pathological diagnosis in 12 of 16 cases (75.0%), whereas the 90-gene expression assay correctly classified 15 of 16 cases (93.8%).

## Discussion

In this study, we developed and validated Deepath-SCC, a deep learning model that predicts the primary site of SCC from routine H&E-stained slides. Trained on five major SCC subtypes, the model achieved an accuracy of 91.2% with a weighted AUROC of 0.986 (95% CI: 0.984–0.989) in the internal test set, and an accuracy of 86.1% in an external test set. In clinically provisional CUP cases, Deepath-SCC showed 75.0% (12/16) concordance with the reference diagnosis, which was lower than that of the 90-gene expression assay (93.8%, 15/16).

SCCs arise from diverse anatomical sites but often share overlapping morphological and immunophenotypic features, posing significant diagnostic challenges for conventional pathology. This is particularly problematic in clinical scenarios involving metastatic SCCs or patients with a history of SCC, where accurate identification of tissue of origin is essential for guiding treatment decisions. The absence of reliable organ-specific markers further complicates routine pathological evaluation of SCC.

Several studies have employed molecular profiling techniques, including gene expression profiles, genomic mutation landscapes and DNA methylation patterns, to trace the tissue of origin in malignancies^9–11^. Nevertheless, these models were primarily established within pan-cancer frameworks and were not specifically optimized for SCCs. Recently, two SCC-specific models based on DNA-methylation profiling have been introduced. Min et al. developed a classifier to distinguish the tumor types of squamous cell carcinoma and urothelial carcinoma, achieving an accuracy of 84.87% in an independent validation cohort^19^. Maximilian et al. showed that a DNA methylation-based classifier could identify the primary sites of head and neck SCC into three anatomical categories: oral cavity, oropharynx, and hypopharynx or larynx, with classification accuracies ranging from 83 to 89%^20^.

Recent computational approaches for tissue-of-origin prediction from WSIs include both task-specific deep learning models and emerging foundation models. Ming et al. proposed TOAD, a convolutional neural network–based framework trained on 18 tumor types, which achieved an overall accuracy of approximately 80% in an external test set and 61% in a CUP cohort; for discrimination among four SCC subtypes, the reported concordance was around 80%^21^. Transformer-based foundation models, such as TITAN, have demonstrated strong general-purpose representation learning in digital pathology; however, dedicated evaluations for SCC-specific tissue-of-origin prediction have not yet been reported^22^. Previously, our group developed a deep learning–based approach for MSI pre-screening directly from WSIs, demonstrating the feasibility of using routine H&E slides for clinically meaningful biomarker assessment^23^. Building on this experience, the present study adopts a similarly task-oriented, end-to-end workflow conceptually aligned with the STAMP framework, which enables biomarker or diagnostic prediction directly from WSIs using deep learning^24^. Rather than adopting a broad pan-cancer strategy, we focused on tissue-of-origin discrimination within pan-SCCs. Using this tailored modeling approach, we observed performance in the range of 86.1–91.2%, suggesting that a task-specific modeling strategy may be feasible for pan-SCC tissue-of-origin prediction.

In addition, we explored a comparison between the 90-gene expression assay and Deepath-SCC for pan-SCC classification. This comparison highlights the complementary strengths of molecular and morphology-driven approaches. The gene expression–based assay showed superior overall accuracy. However, Deepath-SCC model may offer practical advantages in settings where molecular assays are not readily accessible or when tumor tissue is limited, represents a complementary.

The preliminary interpretability analyses provide exploratory insights into the model’s decision-making process for pan-SCC tissue-of-origin prediction. UMAP visualization of high-scoring tiles demonstrated distinct subtype-specific clustering, suggesting that the model captures biologically meaningful morphological patterns. Expert pathologist review further indicated that some discriminative features identified by the model overlap with known histopathological characteristics, including keratinization, bizarre cells, and lymphocytic infiltration. The subtype-specific enrichment of these features supports the biological plausibility of the model and indicates that its predictions are grounded in recognizable pathological cues, thereby enhancing clinical interpretability and confidence. Future work may consider incorporating additional visualization approaches, multimodal inputs (e.g., gene expression data, clinical information, and HPV/EBV status), and emerging explainability frameworks, which could help to better contextualize model predictions and support their evaluation in clinical settings.

This study has several limitations. First, Deepath-SCC was developed and validated primarily using surgical resection specimens, whereas in routine clinical practice, metastatic SCCs are often diagnosed based on biopsy samples. Systematic evaluation of model performance on biopsy-based specimens is therefore required. In addition, the limited size of the CUP cohort restricts statistical power, underscoring the need for validation in larger, multicenter CUP cohorts. Second, the current model includes only five common SCC subtypes and groups non-nasopharyngeal head and neck SCC together with esophageal SCC. Future studies will expand the dataset to include rarer SCC subtypes, such as cutaneous and thymic SCC, and will develop an updated model with finer subtype stratification and broader coverage. Third, additional validation using datasets from more diverse populations, staining protocols, and scanner platforms is necessary to further assess generalizability. Taken together, these limitations indicate that the present findings support the technical feasibility of Deepath-SCC, while substantial further validation will be necessary before expanding its diagnostic applicability.

In summary, this study provides initial evidence for the feasibility of applying deep learning based digital pathology to predict the tissue of origin in pan SCC. Although encouraging performance was observed in retrospective analyses, translation to real-world clinical practice, particularly in diagnostically challenging scenarios such as metastatic SCC or biopsy specimens, will require further validation in larger, multicenter studies.

## Methods

### Study participants

We retrospectively collected H&E-stained slides from formalin-fixed paraffin-embedded (FFPE) tumor tissue blocks archived at FUSCC and CPH affiliated to the Nanjing Medical University. Samples were obtained between January 2015 and December 2024 and comprised five cancer types: CSCC, HNE, LUSCC, NPC, and UC. To enhance population diversity and represent Western cohorts, additional SCC cases were retrieved from TCGA. All dataset partitioning was performed strictly at the patient level to prevent data leakage. WSIs from FUSCC, CPH, and TCGA were pooled and subsequently randomly split at the patient level for model development into training and internal test cohorts, ensuring that no patient contributed slides to more than one subset. The distribution of cases from different centers across the training and internal test sets is summarized in Table S4. An independent external test set comprising the same five SCC subtypes was obtained from AHJU. The study was conducted in accordance with the Declaration of Helsinki and Good Clinical Practice guidelines. Ethics approval was obtained from the Ethics Committee of the lead institution (FUSCC; Approval No. 2309282-22). The requirement for informed consent was waived by the Committee because the study was retrospective and used anonymized data. All WSIs were scanned at 20x magnification and digitized in KFB format (KFBIO, Ningbo, China), MRXS format (3DHISTECH, Budapest, Hungary) or SVS format (Leica, Wetzlar, Germany).

### Image preprocessing and identification of region of interest

**Fig. 5:**
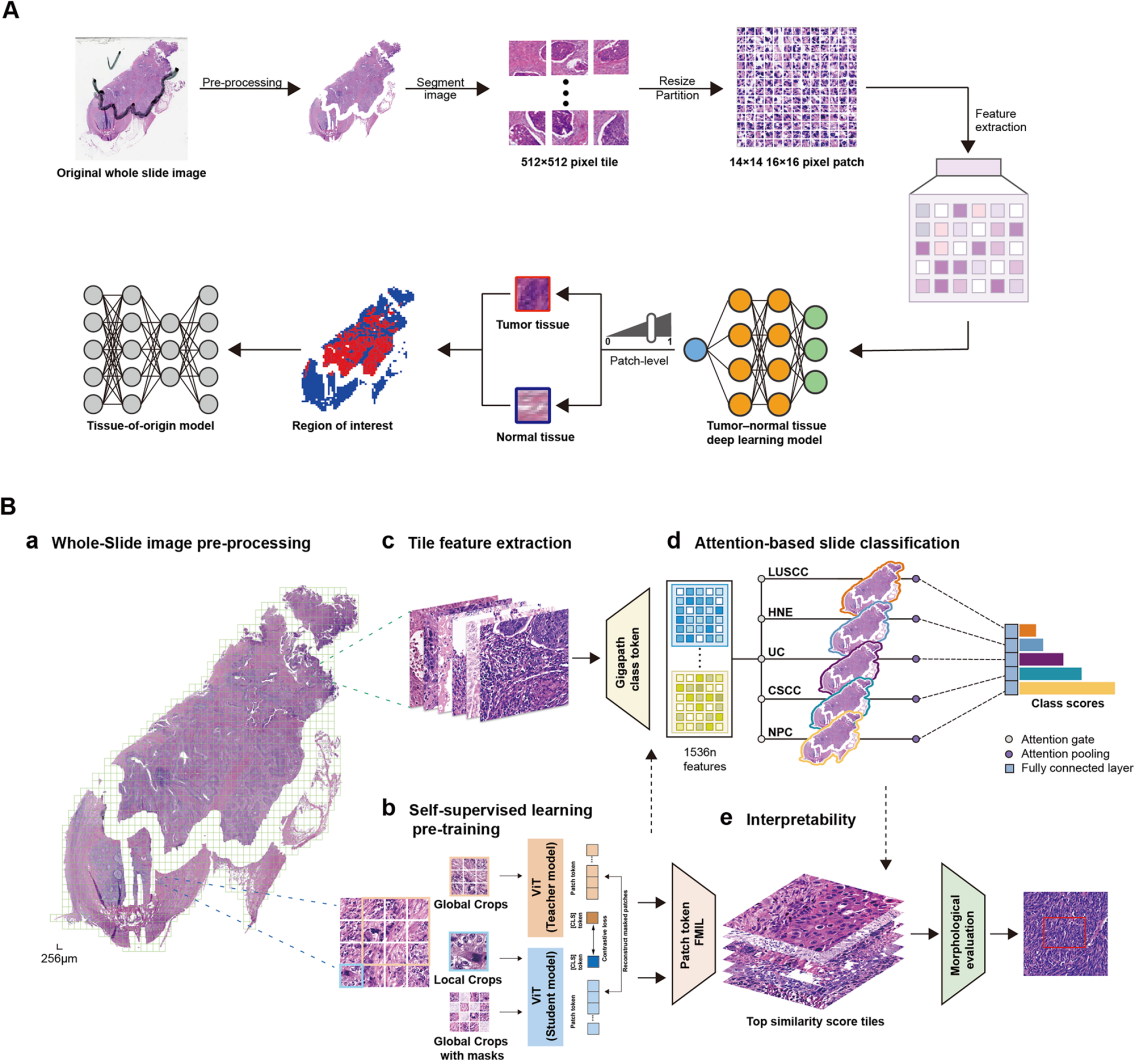
Deepath-SCC workflow overview. **A** Image preprocessing and tumor-normal classification model workflow. **B** Workflow overview of Deepath-SCC.

The image preprocessing pipeline began with the removal of background area, pen markings, and other artifacts from the WSIs. WSIs were then segmented into smaller tiles measuring 256 × 256 μm (at a resolution of 32 μm/pixel). Tiles containing more than 50% background or artifacts were excluded. For the remaining tiles, corresponding high-resolution tiles (512 × 512 pixel at 0.5 μm/pixel) were extracted. These high-resolution tiles were resized to 224 × 224 pixels and further partitioned into 14×14 patches, each measuring 16 × 16 pixel. Each patch was classified as tumor or normal tissue using a pretrained tumor-normal classification model based on Prov-GigaPath. Tiles in which more than 90% of patches were identified as tumor were selected as region of interest (ROI) and used for model training. (Fig. 5A).

### Feature extractor

We developed a feature-based multiple instance learning (FMIL) framework consisting of two primary components: a feature extractor and an aggregation module (Fig. 5B). The feature extractor was pretrained on DINOv2, a state-of-the-art self-supervised learning framework for image analysis^25^.

A persistent challenge in histopathological image analysis is the inconsistency in color and intensity across sections, even within the same institution. These inconsistencies can result from differences in tissue fixation affected by sample size, section thickness due to procedural differences, and the choice of staining reagents during H&E staining. Such variations can interfere with the performance of quantitative image analysis. To mitigate this issue, the feature extractor was pretrained on a large dataset of image tiles, utilizing extensive data augmentation to simulate diverse conditions, and was trained in a fully label-free manner.

Each image tile was independently processed by the feature extractor to generate a compact feature embedding. The feature extractor’s weights were fixed during both training and inference phases, ensuring consistent feature representation throughout the FMIL pipeline.

### Self-supervised learning pretraining

Self-supervised pretraining was implemented using a student-teacher architecture, utilizing momentum-updated teacher weights. In this framework, the teacher network receives global crops of each image, while the student network processes both global and local crops with varying augmentation strengths.

Specifically, global crops are augmented with weaker augmentations, such as random resized cropping and horizontal flipping, while local crops undergo stronger augmentations, including color jittering, Gaussian blurring, and solarization. The difference in augmentation strength promotes learning more nuanced and localized features by the student model.

The total loss function is a weighted combination of the cross-entropy and iBOT losses:

1 $${L}_{total}={L}_{CE}+\lambda {L}_{iBOT}$$

where $\lambda$ balances the two losses.

Cross-Entropy Loss (*L*_*CE*_): This loss encourages local-to-global correspondence. It’s computed between the student’s output for local crops and the sharpened, centered output of the teacher for global crops of the same image:

2 $${L}_{CE}=-\mathop{\sum }\limits_{i=1}^{N}{P}_{t}^{\,globa{l}^{{\prime} }}(i)\log \,{P}_{s}^{local}(i)$$

${P}_{t}^{\,globa{l}^{\mathrm{'}}}$ is the sharpened and centered teacher output for a global crop, and ${P}_{s}^{local}$ is the student output for a local crop. Centering and sharpening are applied to the teacher output:

3 $$C=\frac{1}{T}\mathop{\sum }\limits_{{\rm{t}}=1}^{T}{P}_{t}^{\,global}$$

4 $${P}_{t}^{\,globa{l}^{\mathrm{'}}}=\frac{{P}_{t}^{\,global}-C}{\tau }$$

iBOT Loss (*L*_*iBOT*_): This loss promotes invariance to masking and encourages learning robust, context-aware representations. It’s calculated between the teacher’s output for a global crop and the student’s output for a masked global crop of the same image:

5 $${L}_{iBOT}=-\mathop{\sum }\limits_{{\rm{i}}=1}^{B}\log \frac{\exp (sim({f}_{i}^{t,global},{f}_{i}^{s,maskedglobal})/{\tau }^{{\prime} })}{{\sum }_{j=1}^{B}\exp (sim({f}_{i}^{t,global},{f}_{i}^{s,maskedglobal})/{\tau }^{{\prime} })}$$

Where ${f}_{i}^{t,global}$ is the teacher output for the global crop of image i, and ${f}_{i}^{s,maskedglobal}$ is the student output for the masked global crop of image i. $sim(\cdot ,\cdot )$ denotes cosine similarity, and $\tau {\prime}$ is a temperature parameter. The pretrained encoder resulting from minimizing this combined loss is then used to initialize the encoder of our segmentation network.

### Deepath-SCC classifier

The Deepath-SCC model was implemented using PyTorch and trained on an NVIDIA RTX 4090 GPU with 24 GB of memory^25^. The Deepath-SCC classifier was developed using an MIL framework, following methods previously established for handling WSI data^26,27^. Unlike approaches that rely on sub-sampling a fixed number of tiles, MIL utilizes all tiles from a patient’s WSI as a “bag” without assuming every tile within the bag directly reflects the SCC types, which allows for greater resilience to intratumor heterogeneity. Furthermore, an attention mechanism was incorporated within the decoder, providing access to the full scope of encoded information. This mechanism assigns variable attention weights to different input regions, identifying the significance of each token and allocating priority for the generation of output tokens at every step.

### The 90-gene expression assay

To objectively assess the performance of the model in CUP, we retrospectively collected provisional CUP cases that had undergone testing with the 90-gene expression assay (Canhelp-Origin Assay, Canhelp Genomics Co., Ltd., Hangzhou, China). All cases were identified through the multidisciplinary team (MDT) for cancers of multiple and unknown primaries at FUSCC. The final reference diagnosis for each case was established according to a predefined CUP diagnostic workflow, integrating standard diagnostic evaluations, including imaging studies, histopathological assessment, molecular findings, and follow-up when available^6^.

The 90-gene expression assay is a gene expression profiling-based test that identifies 21 common tumor types using quantitative reverse transcription polymerase chain reaction (RT-qPCR) on total RNA isolated from FFPE tumor tissue, which obtained the Conformité Européenne mark and approval from the National Medical Products Administration in China^9^. For each case, a predefined 90-gene classifier was utilized to analyze the gene expression patterns and generate similarity scores for all 21 tumor types. These scores are probability-based, each ranging from 0 to 100, and together summing to 100 for each sample. The tumor type with the highest similarity score was considered the predicated tumor type.

### Implementation

Tumor regions identified by the model were reviewed and validated by two experienced pathologists to ensure the reliability of tile selection. Feature extraction was performed using Prov-GigaPath, from which both patch-level tokens and class tokens were obtained for each tile. Patch-level tokens were used to train the tumor–normal classification model, while class tokens were leveraged for training the Deepath-SCC model. For network training, the model was trained on the training dataset for 200 epochs with a batch size of 16. A cosine annealing strategy was adopted for learning rate scheduling, and the AdamW optimizer was used with an initial learning rate of 1e-4 and a weight decay of 1e-5.

### Statistical analysis

AUROC and accuracy were used as the primary metric to measure classification performance. The optimal similarity score threshold was determined by optimal binning algorithm in the SPSS software (version 26.0). Statistical analysis was performed with R software (version 4.4.3).

## Supplementary information

Supplementary Information

## Data Availability

To protect patient privacy, pathology image datasets and other patient-related information of the collected in-house datasets are not publicly available. However, all these data can be accessed upon reasonable request by contacting the corresponding author via email. High-resolution diagnostic whole slide image data from TCGA, as well as the associated diagnoses, can be publicly accessed via the National Institutes of Health Genomic Data Commons.

## Code availability

The code used in this study is publicly available at Zenodo: https://zenodo.org/records/18184207.
