# Supplementary material for: Deepath-SCC: a deep learning model for accurate tissue origin identification in squamous cell carcinoma
Source: NPJ Precis Oncol. 2026 Apr 10;10:214. doi: 10.1038/s41698-026-01405-1 (PMC13254084; doi:10.1038/s41698-026-01405-1)

Supplementary material

Contents

Table S1. Model performance across different tile thresholds 2

Table S2. Proportion of 11 histological features across five SCC subtypes 9

Table S3. Clinicopathological features of 16 cases of provisional cancer of unknown primary 10

Table S4. Distribution of cases across train and internal test set by data source 11

Figure S1. Classification performance of the model across varying tile numbers in the internal test set 12

Figure S2. Confusion matrices under different filtering criteria in the internal test set 13

Table S1. Model performance across different tile thresholds

Group	Cancer	Tiles ≥ 10							
		Total	Correct	ROC	95%CI	Accuracy	95%CI	PPV	95%CI
All	CSCC	544	497	0.987	0.983 ~ 0.991	91.4%	88.7% ~ 93.4%	98.8%	97.4% ~ 99.5%
	HNE	431	390	0.979	0.973 ~ 0.986	90.5%	87.3% ~ 92.9%	87.8%	84.5% ~ 90.6%
	LUSCC	417	376	0.984	0.978 ~ 0.990	90.2%	86.9% ~ 92.7%	89.5%	86.2% ~ 92.1%
	NPC	64	60	0.989	0.971 ~ 1.000	93.8%	85.0% ~ 97.5%	75.9%	65.5% ~ 84.0%
	UC	307	279	0.988	0.984 ~ 0.993	90.9%	87.1% ~ 93.6%	88.3%	84.3% ~ 91.4%
	Total	1763	1602	0.985	0.982 ~ 0.988	90.9%	89.4% ~ 92.1%	88.1%	85.8% ~ 90.2%
Primary	CSCC	433	400	0.989	0.985 ~ 0.994	92.4%	89.5% ~ 94.5%	99.0%	97.5% ~ 99.6%
	HNE	394	362	0.984	0.979 ~ 0.990	91.9%	88.8% ~ 94.2%	89.8%	86.5% ~ 92.4%
	LUSCC	391	357	0.988	0.984 ~ 0.993	91.3%	88.1% ~ 93.7%	91.3%	88.1% ~ 93.7%
	NPC	42	39	0.985	0.959 ~ 1.000	92.9%	81.0% ~ 97.5%	76.5%	63.2% ~ 86.0%
	UC	289	265	0.989	0.984 ~ 0.993	91.7%	87.9% ~ 94.4%	88.6%	84.5% ~ 91.7%
	Total	1549	1423	0.988	0.986 ~ 0.991	91.9%	90.4% ~ 93.1%	89.0%	86.4% ~ 91.5%
Metastatic	CSCC	111	97	0.964	0.941 ~ 0.986	87.4%	79.9% ~ 92.3%	98.0%	92.9% ~ 99.4%
	HNE	37	28	0.914	0.860 ~ 0.967	75.7%	59.9% ~ 86.6%	68.3%	53.0% ~ 80.4%
	LUSCC	26	19	0.909	0.841 ~ 0.977	73.1%	53.9% ~ 86.3%	65.5%	47.3% ~ 80.1%
	NPC	22	21	0.993	0.982 ~ 1.000	95.5%	78.2% ~ 99.2%	75.0%	56.6% ~ 87.3%
	UC	18	14	0.978	0.952 ~ 1.000	77.8%	54.8% ~ 91.0%	82.4%	59.0% ~ 93.8%
	Total	214	179	0.956	0.938 ~ 0.971	83.6%	78.1% ~ 88.0%	77.8%	70.7% ~ 84.2%

Table S1. Model performance across different tile thresholds

Group	Cancer	Tiles ≥ 20							
		Total	Correct	ROC	95%CI	Accuracy	95%CI	PPV	95%CI
All	CSCC	528	485	0.988	0.984 ~ 0.992	91.9%	89.2% ~ 93.9%	99.0%	97.6% ~ 99.6%
	HNE	402	365	0.981	0.974 ~ 0.987	90.8%	87.6% ~ 93.2%	88.0%	84.5% ~ 90.7%
	LUSCC	397	360	0.985	0.980 ~ 0.991	90.7%	87.4% ~ 93.2%	89.6%	86.2% ~ 92.2%
	NPC	59	55	0.988	0.969 ~ 1.000	93.2%	83.8% ~ 97.3%	78.6%	67.6% ~ 86.6%
	UC	276	251	0.989	0.984 ~ 0.994	90.9%	87.0% ~ 93.8%	88.4%	84.1% ~ 91.6%
	Total	1662	1516	0.986	0.983 ~ 0.989	91.2%	89.8% ~ 92.5%	88.7%	86.2% ~ 90.8%
Primary	CSCC	424	393	0.990	0.986 ~ 0.994	92.7%	89.8% ~ 94.8%	99.0%	97.4% ~ 99.6%
	HNE	367	337	0.986	0.980 ~ 0.991	91.8%	88.6% ~ 94.2%	90.1%	86.7% ~ 92.7%
	LUSCC	371	341	0.990	0.986 ~ 0.993	91.9%	88.7% ~ 94.3%	90.9%	87.6% ~ 93.4%
	NPC	38	35	0.984	0.955 ~ 1.000	92.1%	79.2% ~ 97.3%	79.5%	65.5% ~ 88.8%
	UC	260	238	0.989	0.984 ~ 0.994	91.5%	87.5% ~ 94.3%	88.5%	84.1% ~ 91.8%
	Total	1460	1344	0.989	0.987 ~ 0.992	92.1%	90.6% ~ 93.3%	89.6%	86.8% ~ 92.2%
Metastatic	CSCC	104	92	0.969	0.949 ~ 0.990	88.5%	80.9% ~ 93.3%	98.9%	94.2% ~ 99.8%
	HNE	35	28	0.920	0.866 ~ 0.975	80.0%	64.1% ~ 90.0%	68.3%	53.0% ~ 80.4%
	LUSCC	26	19	0.915	0.848 ~ 0.982	73.1%	53.9% ~ 86.3%	70.4%	51.5% ~ 84.1%
	NPC	21	20	0.992	0.981 ~ 1.000	95.2%	77.3% ~ 99.2%	76.9%	57.9% ~ 89.0%
	UC	16	13	0.990	0.975 ~ 1.000	81.3%	57.0% ~ 93.4%	86.7%	62.1% ~ 96.3%
	Total	202	172	0.959	0.940 ~ 0.976	85.1%	79.6% ~ 89.4%	80.2%	73.3% ~ 86.7%

Table S1. Model performance across different tile thresholds

Group	Cancer	Tiles ≥ 30							
		Total	Correct	ROC	95%CI	Accuracy	95%CI	PPV	95%CI
All	CSCC	514	472	0.988	0.983 ~ 0.992	91.8%	89.1% ~ 93.9%	99.2%	97.9% ~ 99.7%
	HNE	377	343	0.983	0.977 ~ 0.988	91.0%	87.7% ~ 93.5%	87.7%	84.1% ~ 90.6%
	LUSCC	385	349	0.984	0.979 ~ 0.990	90.6%	87.3% ~ 93.2%	89.5%	86.0% ~ 92.2%
	NPC	56	53	0.989	0.968 ~ 1.000	94.6%	85.4% ~ 98.2%	79.1%	67.9% ~ 87.1%
	UC	252	228	0.988	0.983 ~ 0.993	90.5%	86.2% ~ 93.5%	88.0%	83.5% ~ 91.4%
	Total	1584	1445	0.987	0.984 ~ 0.989	91.2%	89.7% ~ 92.5%	88.7%	86.4% ~ 90.9%
Primary	CSCC	418	388	0.990	0.986 ~ 0.994	92.8%	89.9% ~ 94.9%	99.2%	97.8% ~ 99.7%
	HNE	345	317	0.986	0.981 ~ 0.991	91.9%	88.5% ~ 94.3%	90.1%	86.5% ~ 92.8%
	LUSCC	360	330	0.989	0.985 ~ 0.993	91.7%	88.4% ~ 94.1%	90.9%	87.5% ~ 93.5%
	NPC	35	33	0.983	0.951 ~ 1.000	94.3%	81.4% ~ 98.4%	78.6%	64.1% ~ 88.3%
	UC	238	217	0.988	0.983 ~ 0.993	91.2%	86.9% ~ 94.2%	87.9%	83.2% ~ 91.4%
	Total	1396	1285	0.989	0.987 ~ 0.991	92.0%	90.5% ~ 93.4%	89.3%	86.5% ~ 92.1%
Metastatic	CSCC	96	84	0.966	0.943 ~ 0.989	87.5%	79.4% ~ 92.7%	98.8%	93.6% ~ 99.8%
	HNE	32	26	0.938	0.903 ~ 0.973	81.3%	64.7% ~ 91.1%	66.7%	51.0% ~ 79.4%
	LUSCC	25	19	0.913	0.842 ~ 0.984	76.0%	56.6% ~ 88.5%	70.4%	51.5% ~ 84.1%
	NPC	21	20	0.997	0.992 ~ 1.000	95.2%	77.3% ~ 99.2%	80.0%	60.9% ~ 91.1%
	UC	14	11	0.990	0.974 ~ 1.000	78.6%	52.4% ~ 92.4%	91.7%	64.6% ~ 98.5%
	Total	188	160	0.962	0.944 ~ 0.977	85.1%	79.3% ~ 89.5%	81.5%	74.3% ~ 87.5%

Table S1. Model performance across different tile thresholds

Group	Cancer	Tiles ≥ 50							
		Total	Correct	ROC	95%CI	Accuracy	95%CI	PPV	95%CI
All	CSCC	502	464	0.989	0.985 ~ 0.993	92.4%	89.8% ~ 94.4%	99.1%	97.8% ~ 99.7%
	HNE	335	306	0.986	0.98 ~ 0.991	91.3%	87.8% ~ 93.9%	89.0%	85.2% ~ 91.8%
	LUSCC	354	324	0.985	0.979 ~ 0.992	91.5%	88.2% ~ 94.0%	89.5%	85.9% ~ 92.3%
	NPC	49	47	0.999	0.998 ~ 1.000	95.9%	86.3% ~ 98.9%	78.3%	66.4% ~ 86.9%
	UC	219	199	0.990	0.986 ~ 0.994	90.9%	86.3% ~ 94.0%	88.4%	83.6% ~ 92.0%
	Total	1459	1340	0.988	0.986 ~ 0.991	91.8%	90.3% ~ 93.1%	88.9%	86.4% ~ 91.2%
Primary	CSCC	413	385	0.991	0.988 ~ 0.995	93.2%	90.4% ~ 95.3%	99.2%	97.8% ~ 99.7%
	HNE	304	281	0.989	0.984 ~ 0.994	92.4%	88.9% ~ 94.9%	91.2%	87.5% ~ 93.9%
	LUSCC	331	307	0.990	0.987 ~ 0.994	92.7%	89.4% ~ 95.1%	91.1%	87.6% ~ 93.7%
	NPC	28	27	0.999	0.998 ~ 1.000	96.4%	82.3% ~ 99.4%	77.1%	61.0% ~ 87.9%
	UC	207	190	0.990	0.986 ~ 0.995	91.8%	87.2% ~ 94.8%	88.4%	83.4% ~ 92.0%
	Total	1283	1190	0.991	0.989 ~ 0.993	92.8%	91.2% ~ 94.0%	89.4%	86.2% ~ 92.4%
Metastatic	CSCC	89	79	0.967	0.944 ~ 0.991	88.8%	80.5% ~ 93.8%	98.8%	93.3% ~ 99.8%
	HNE	31	25	0.937	0.901 ~ 0.974	80.6%	63.7% ~ 90.8%	69.4%	53.1% ~ 82.0%
	LUSCC	23	17	0.907	0.832 ~ 0.983	73.9%	53.5% ~ 87.5%	68.0%	48.4% ~ 82.8%
	NPC	21	20	0.997	0.992 ~ 1.000	95.2%	77.3% ~ 99.2%	80.0%	60.9% ~ 91.1%
	UC	12	9	0.991	0.976 ~ 1.000	75.0%	46.8% ~ 91.1%	90.0%	59.6% ~ 98.2%
	Total	176	150	0.962	0.943 ~ 0.978	85.2%	79.2% ~ 89.7%	81.2%	73.8% ~ 87.8%

Table S1. Model performance across different tile thresholds

Group	Cancer	Tiles ≥ 70							
		Total	Correct	ROC	95%CI	Accuracy	95%CI	PPV	95%CI
All	CSCC	489	453	0.989	0.984 ~ 0.993	92.6%	90.0% ~ 94.6%	99.1%	97.8% ~ 99.7%
	HNE	305	281	0.987	0.982 ~ 0.992	92.1%	88.6% ~ 94.7%	88.9%	85.0% ~ 91.9%
	LUSCC	320	295	0.986	0.979 ~ 0.992	92.2%	88.7% ~ 94.7%	89.7%	85.9% ~ 92.5%
	NPC	41	39	0.999	0.998 ~ 1.000	95.1%	83.9% ~ 98.7%	78.0%	64.8% ~ 87.2%
	UC	196	176	0.990	0.986 ~ 0.994	89.8%	84.8% ~ 93.3%	88.4%	83.3% ~ 92.2%
	Total	1351	1244	0.989	0.986 ~ 0.991	92.1%	90.5% ~ 93.4%	88.8%	85.9% ~ 91.5%
Primary	CSCC	407	380	0.991	0.987 ~ 0.995	93.4%	90.5% ~ 95.4%	99.2%	97.7% ~ 99.7%
	HNE	274	256	0.991	0.987 ~ 0.996	93.4%	89.9% ~ 95.8%	91.4%	87.6% ~ 94.2%
	LUSCC	298	279	0.992	0.988 ~ 0.995	93.6%	90.3% ~ 95.9%	91.2%	87.5% ~ 93.9%
	NPC	21	20	0.999	0.997 ~ 1.000	95.2%	77.3% ~ 99.2%	76.9%	57.9% ~ 89.0%
	UC	185	168	0.990	0.985 ~ 0.994	90.8%	85.8% ~ 94.2%	88.4%	83.1% ~ 92.2%
	Total	1185	1103	0.992	0.989 ~ 0.994	93.1%	91.5% ~ 94.4%	89.4%	85.8% ~ 92.9%
Metastatic	CSCC	82	73	0.966	0.941 ~ 0.991	89.0%	80.4% ~ 94.1%	98.6%	92.7% ~ 99.8%
	HNE	31	25	0.935	0.896 ~ 0.973	80.6%	63.7% ~ 90.8%	69.4%	53.1% ~ 82.0%
	LUSCC	22	16	0.903	0.826 ~ 0.981	72.7%	51.8% ~ 86.8%	69.6%	49.1% ~ 84.4%
	NPC	20	19	0.996	0.991 ~ 1.000	95.0%	76.4% ~ 99.1%	79.2%	59.5% ~ 90.8%
	UC	11	8	0.992	0.976 ~ 1.000	72.7%	43.4% ~ 90.3%	88.9%	56.5% ~ 98.0%
	Total	166	141	0.960	0.941 ~ 0.977	84.9%	78.7% ~ 89.6%	81.1%	73.5% ~ 87.9%

Table S1. Model performance across different tile thresholds

Group	Cancer	Tiles ≥ 100							
		Total	Correct	ROC	95%CI	Accuracy	95%CI	PPV	95%CI
All	CSCC	473	440	0.99	0.985 ~ 0.994	93%	90.4% ~ 95.0%	99.1%	97.7% ~ 99.6%
	HNE	264	246	0.989	0.984 ~ 0.994	93.2%	89.5% ~ 95.6%	88.8%	84.6% ~ 92.0%
	LUSCC	292	270	0.987	0.980 ~ 0.994	92.5%	88.9% ~ 95.0%	90.6%	86.8% ~ 93.4%
	NPC	35	34	0.999	0.998 ~ 1.000	97.1%	85.5% ~ 99.5%	81.0%	66.7% ~ 90.0%
	UC	164	148	0.990	0.985 ~ 0.995	90.2%	84.7% ~ 93.9%	88.6%	82.9% ~ 92.6%
	Total	1228	1138	0.990	0.987 ~ 0.992	92.7%	91.1% ~ 94.0%	89.6%	86.7% ~ 92.2%
Primary	CSCC	399	373	0.991	0.987 ~ 0.995	93.5%	90.6% ~ 95.5%	99.2%	97.7% ~ 99.7%
	HNE	238	225	0.992	0.987 ~ 0.996	94.5%	90.9% ~ 96.8%	91.1%	86.9% ~ 94.0%
	LUSCC	270	254	0.993	0.99 ~ 0.996	94.1%	90.6% ~ 96.3%	92.0%	88.2% ~ 94.7%
	NPC	19	18	0.999	0.997 ~ 1.000	94.7%	75.4% ~ 99.1%	78.3%	58.1% ~ 90.3%
	UC	155	141	0.989	0.984 ~ 0.995	91.0%	85.4% ~ 94.5%	88.7%	82.8% ~ 92.7%
	Total	1081	1011	0.992	0.99 ~ 0.994	93.5%	91.9% ~ 94.8%	89.9%	85.9% ~ 93.2%
Metastatic	CSCC	74	67	0.972	0.949 ~ 0.994	90.5%	81.7% ~ 95.3%	98.5%	92.1% ~ 99.7%
	HNE	26	21	0.942	0.904 ~ 0.98	80.8%	62.1% ~ 91.5%	70.0%	52.1% ~ 83.3%
	LUSCC	22	16	0.901	0.824 ~ 0.979	72.7%	51.8% ~ 86.8%	72.7%	51.8% ~ 86.8%
	NPC	16	16	0.997	0.991 ~ 1.000	100.0%	80.6% ~ 100%	84.2%	62.4% ~ 94.5%
	UC	9	7	0.992	0.975 ~ 1.000	77.8%	45.3% ~ 93.7%	87.5%	52.9% ~ 97.8%
	Total	147	127	0.964	0.945 ~ 0.981	86.4%	79.9% ~ 91.0%	82.6%	74.3% ~ 89.6%

Table S1. Model performance across different tile thresholds

Group	Cancer	Tiles ≥ 20 and similarity score ≥ 0.7914							
		Total	Correct	ROC	95%CI	Accuracy	95%CI	PPV	95%CI
All	CSCC	460	447	0.995	0.992 ~ 0.998	97.2%	95.2% ~ 98.3%	99.6%	98.4% ~ 99.9%
	HNE	325	309	0.990	0.984 ~ 0.996	95.1%	92.2% ~ 96.9%	93.9%	90.8% ~ 96.0%
	LUSCC	331	315	0.995	0.990 ~ 0.999	95.2%	92.3% ~ 97.0%	96.0%	93.3% ~ 97.7%
	NPC	55	52	0.990	0.972 ~ 1.000	94.5%	85.1% ~ 98.1%	88.1%	77.5% ~ 94.1%
	UC	214	209	0.996	0.992 ~ 0.999	97.7%	94.6% ~ 99.0%	95.0%	91.3% ~ 97.2%
	Total	1385	1332	0.993	0.990 ~ 0.995	96.2%	95.0% ~ 97.1%	94.5%	92.6% ~ 96.3%
Primary	CSCC	375	365	0.996	0.993 ~ 0.998	97.3%	95.2% ~ 98.5%	99.7%	98.5% ~ 100.0%
	HNE	306	292	0.993	0.989 ~ 0.997	95.4%	92.5% ~ 97.3%	94.8%	91.7% ~ 96.8%
	LUSCC	317	303	0.996	0.994 ~ 0.999	95.6%	92.7% ~ 97.4%	95.9%	93.1% ~ 97.6%
	NPC	38	35	0.987	0.962 ~ 1.000	92.1%	79.2% ~ 97.3%	89.7%	76.4% ~ 95.9%
	UC	205	201	0.996	0.992 ~ 0.999	98.0%	95.1% ~ 99.2%	94.8%	90.9% ~ 97.1%
	Total	1241	1196	0.995	0.993 ~ 0.997	96.4%	95.2% ~ 97.3%	95.0%	92.7% ~ 96.9%
Metastatic	CSCC	85	82	0.990	0.976 ~ 1.000	96.5%	90.1% ~ 98.8%	98.8%	93.5% ~ 99.8%
	HNE	19	17	0.943	0.859 ~ 1.000	89.5%	68.6% ~ 97.1%	81.0%	60.0% ~ 92.3%
	LUSCC	14	12	0.957	0.874 ~ 1.000	85.7%	60.1% ~ 96.0%	100.0%	75.8% ~ 100.0%
	NPC	17	17	0.992	0.978 ~ 1.000	100.0%	81.6% ~ 100.0%	85.0%	64.0% ~ 94.8%
	UC	9	8	0.993	0.980 ~ 1.000	88.9%	56.5% ~ 98.0%	100.0%	67.6% ~ 100.0%
	Total	144	136	0.977	0.957 ~ 0.993	94.4%	89.4% ~ 97.2%	92.9%	88.0% ~ 97.4%

Table S2. Proportion of 11 histological features across five SCC subtypes. P-values were calculated using Fisher's exact test to evaluate differences across tumor groups.

Characteristics	CSCC n=20	HNE n=20	LUSCC n=20	NPC n=20	UC n=20	*P* value
Necrosis	1	0	6	0	0	0.009
Bizarre cell	0	1	16	2	4	<0.001
Koilocytosis	5	6	0	0	0	<0.001
Mitotic figures	4	3	14	1	0	<0.001
Keratinization	2	12	3	0	3	<0.001
Differentiation	3	16	1	0	5	<0.001
Intercellular bridge	1	17	1	0	0	<0.001
Neutrophilic infiltration	5	0	10	4	1	<0.001
Lymphocytic infiltration	3	7	11	20	4	<0.001
Basaloid cell arrangement	1	9	0	0	5	<0.001
Desmoplastic stromal reaction	17	3	9	1	2	<0.001

Table S3. Clinicopathological features of 16 cases of provisional cancer of unknown primary

ID	Age	Sex	Sampling site	Sampling method	Tumor differentiation	Reference Diagnosis	Deepath-SCC		90-gene expression assay	
							Tumor type	Similarity score	Tumor type	Similarity score
1	74	Male	Left supraclavicular lymph node	Surgery	Poorly	UC	UC	93.5	UC	94.7
2	60	Male	Right cervical lymph node	Biopsy	Unknown	OSCC	HNE	44.9	HNSCC	55.4
3	61	Male	Left cervical lymph node	Surgery	Poorly	OSCC	HNE	60.2	HNSCC	64.9
4	56	Male	Right cervical lymph node	Surgery	Unknown	TSCC	NPC	79.2	HNSCC	51.6
5	54	Male	Peritoneum	Surgery	Poorly	UC	UC	50.6	UC	44.0
6	66	Male	Right cervical lymph node	Surgery	Poorly	NPC	NPC	99.5	HNSCC	74.5
7	74	Male	Right lung	Surgery	Poorly	LUSCC	LUSCC	94.2	LUSCC	51.7
8	74	Male	Left lung	Surgery	Poorly	LUSCC	LUSCC	100.0	LUSCC	97.6
9	52	Female	Abdominal cavity	Biopsy	Poorly	CSCC	CSCC	98.9	CSCC	91.3
10	73	Male	Right cervical lymph node	Biopsy	Poorly	NPC	NPC	99.9	HNSCC	97.7
11	39	Male	Right cervical lymph node	Biopsy	Poorly	NPC	NPC	99.3	HNSCC	86.4
12	63	Male	Mesentery	Surgery	Poorly	UC	NPC	44.0	Other	62.4
13	71	Male	Right cervical lymph node	Biopsy	Poorly	UC	HNE	59.0	UC	93.4
14	60	Female	Right Iliac Artery	Surgery	Unknown	CSCC	CSCC	76.2	CSCC	83.8
15	52	Female	Pelvic lymph nodes	Surgery	Unknown	CSCC	CSCC	99.5	CSCC	93.9
16	58	Female	Left inguinal region	Surgery	Unknown	CSCC	HNE	76.6	CSCC	97.0

*Abbreviation: CSCC, cervical squamous cell carcinoma; HNSCC, head and neck squamous cell carcinoma (including nasopharyngeal carcinoma); HNE, head and neck or esophageal squamous cell carcinoma (excluding nasopharyngeal carcinoma); LUSCC, lung squamous cell carcinoma; NPC, nasopharyngeal carcinoma; OSCC, oropharyngeal squamous cell carcinoma; TSCC, tonsil squamous cell carcinoma; UC, urothelial carcinoma.

Table S4. Distribution of cases across train and internal test set by data source.

Type	Training set n=2,267				Internal test set n=1,950			
	FUSCC	CPH	TCGA	Total	FUSCC	CPH	TCGA	Total
CSCC	703	82	69	854	446	79	54	579
HNE	337	0	153	490	238	0	228	466
LUSCC	288	0	198	486	176	0	277	453
NPC	121	0	0	121	77	0	0	77
UC	172	0	144	316	119	0	256	375
Total	1621	82	564	2,267	1,056	79	815	1,950

Figure S1. Classification performance of the model across varying tile numbers in the internal test set.

Confusion matrices showing precision and recall for five SCC types at tile thresholds of (A) 10, (B) 20, (C) 30, (D) 50, (E) 70, and (F) 100 in the test set. One-vs-rest ROC curves for each SCC type. Bar plots depict top-n (n=1,2,3) accuracy for tumor origin classification.

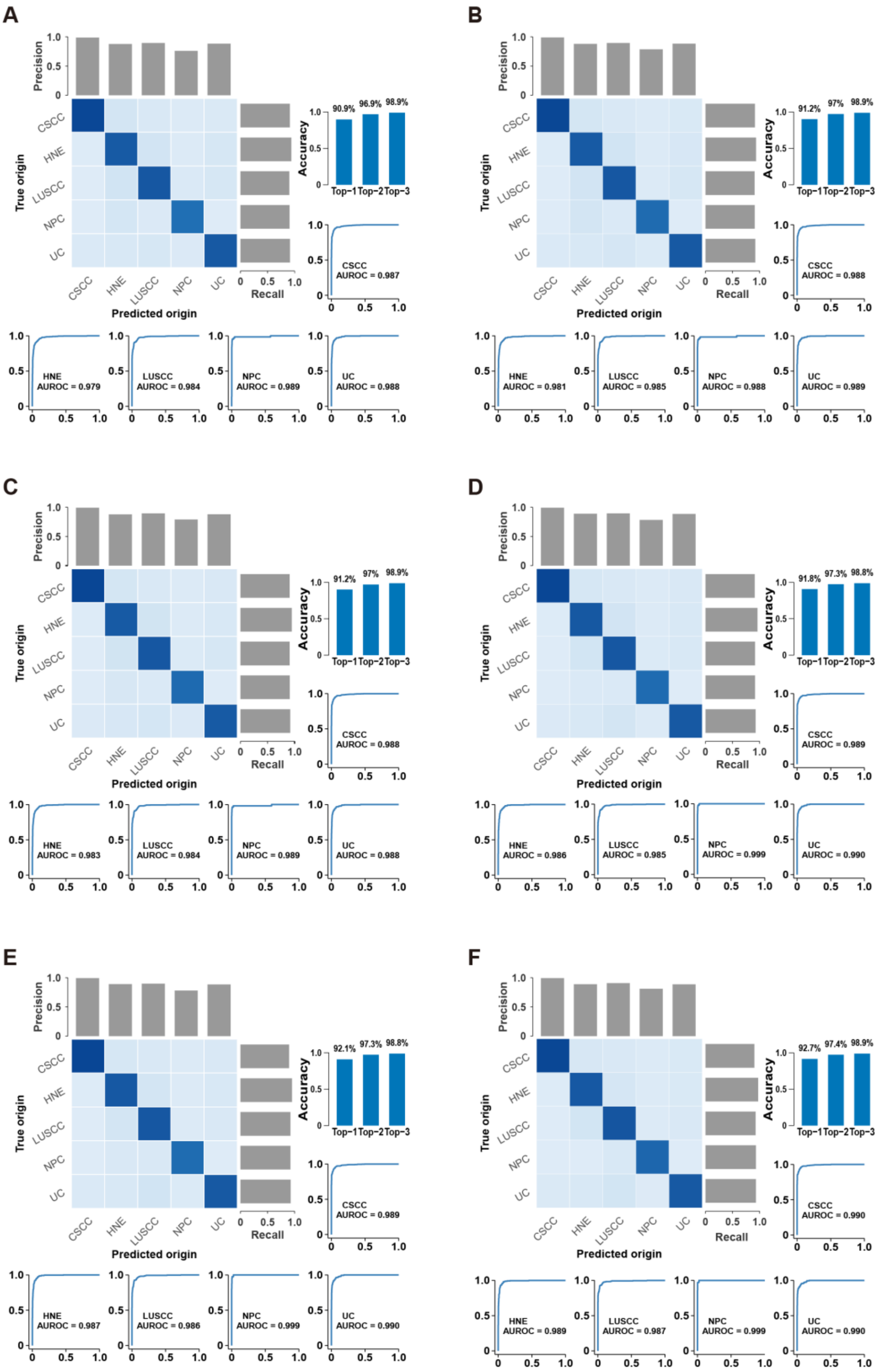

Figure S2. Confusion matrices under different filtering criteria in the internal test set.

(A) Tumor tiles ≥ 20. (B) Tumor tiles ≥ 20 and maximum similarity score ≥ 0.7914.

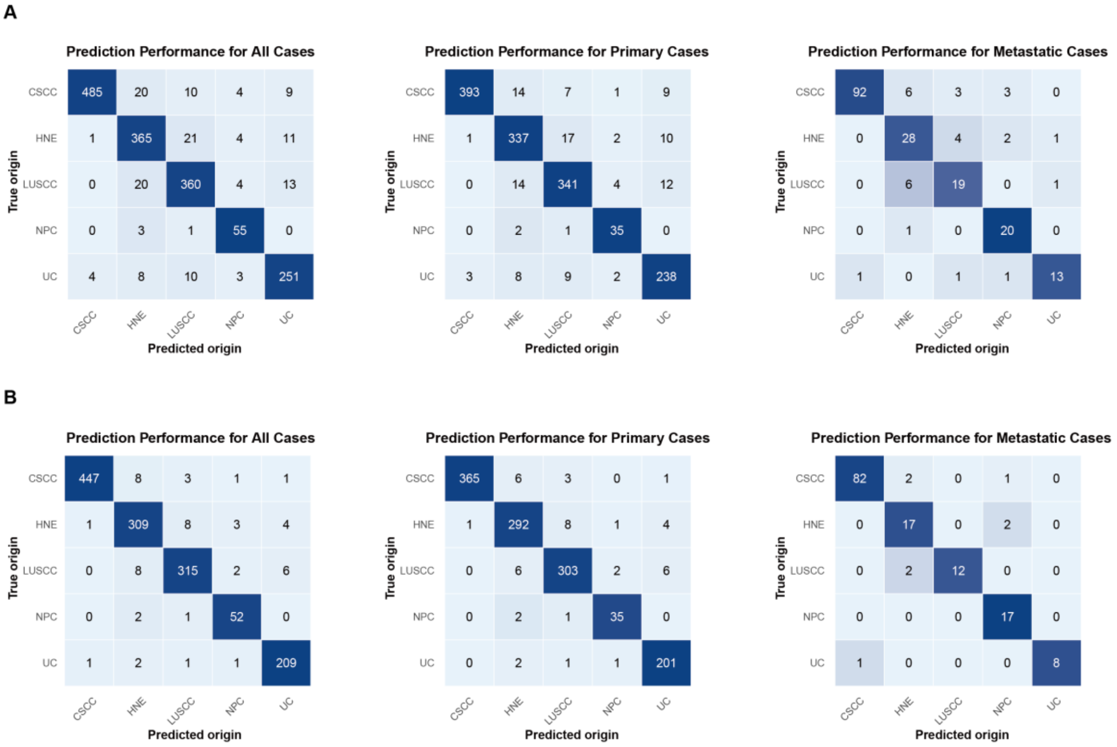

Supplement: Supplementary file 1 — Supplementary Information [file 41698_2026_1405_MOESM1_ESM.pdf]
